# Supplementary material for: Ergodic seismic precursors and transfer learning for short term eruption forecasting at data scarce volcanoes
Source: Nat Commun. 2025 Feb 25;16:1758. doi: 10.1038/s41467-025-56689-x (PMC11861682; doi:10.1038/s41467-025-56689-x)
Supplement: Supplementary file 1 — Supplementary Information [file 41467_2025_56689_MOESM1_ESM.pdf]

SUPPLEMENTARY MATERIAL

Supplementary Tables

Table S1. AUC and AOC values for the forecasting models depicted in Figure 3 (ML: Machine Learning; RSAM: Real Time Seismic Amplitude Measurement for 6 hours average). Forecaster models include tailored models for Whakaari (Whak.), Bezymianny (Bezy.), Copahue (Cop.), as well as Generalized Magmatic (Magma.), Phreatic (Phrea.), and World pools. The "Mean" column is the average AUC across all models.

(a) AUC	Whak.	Bezy.	Cop.	Magma.	Phrea.	World	Mean
ML tailored	0.89	0.77	0.96				0.87
ML Gen CVV	0.96	0.75	0.93	0.81	0.80	0.80	0.84
RSAM 6h	0.86	0.70	0.76	0.50	0.74	0.58	0.69

(b) AOC	Whakaari	Bezymianny	Copahue	Magmatic	Phreatic	World	Mean
ML tailored	0.11	0.23	0.04				0.13
ML Gen CVV	0.04	0.25	0.07	0.19	0.20	0.20	0.16
RSAM 6h	0.14	0.30	0.24	0.50	0.26	0.42	0.31

Table S2. Basic information on volcanoes included in this study indicating the country, the station and network used and its distance to the crater, the number of eruptions recorded, the type and year of eruptions, the length of the seismic record analysed, and the % of continuous data on the record. The last column indicates the number of eruptions anticipated based on whether any model predicted the eruption to be in a high state.

Volcano	***Country***	***Station***	***Network***	***# erup.***	***Type of erup.***	***Eruptions year***	***Record years***	***Eruptions anticipated***
**Pavlof**	Alaska, USA	PVV 4 km	AV	3	Magmatic	14, 14	2	**0/2**
**Veniaminof**	Alaska, USA	VNSS 5.3 km	AV	2	Magmatic	13,18	4	**2/2**
**Bezymianny**	Kamchatka, Russia	BELO 1 km	YC	3	Magmatic	07,08,09	1	**3/3**
**Whakaari**	New Zealand	WIZ 500 m	NZ	5	Phreatic	12,13,13, 16,19	11	**4/5**
**Tongariro**	New Zealand	KRVZ 2 km	NZ	2	Phreatic	12,12	14	**0/2**
**Ruapehu**	New Zealand	FWVZ 2.5 km	NZ	3	Phreatic	06,07	14	1/2
**Redoubt**	Alaska, USA	REF 2.5 km	AV	1	Magmatic	09	.3	**1/1**
**Augustine**	Alaska, USA	AUH 1 km	AV	1	Magmatic	06	1	**1/1**
**Great Sitkin**	Alaska, USA	GSTR 4.5 km	AV	3	Magmatic	21	2	**1/1**
**Semisipochnpo**	Alaska, USA	CETU 7 km	AV	2	Magmatic	19 (2)	.5	**2/2**
**Okmok**	Alaska, USA	OKWR 5 km	AV	1	Magmatic	08	1	**1/1**
**St Helens**	USA	SHW 1 km	AV	1	Magmatic	04	1	**1/1**
**Telica**	Nicaragua	TBTN 0.5 km	6D	3	Magmatic	11,12,13	2	-
**Poas**	Costa Rica	CRPO 0.3 km	OV	56			1	-
**Turrialba**	Costa Rica	VTUN 0.2 km	OV	2		14,15	1.5	-
**Rincon de la Vieja**	Costa Rica	VRLE 2 km	OV	3		14,15,17	3	-
**Montserrat**	UK	MBGH 3.6 km	NA	2		04,05	2	-
**Eyjafjallajökull**	Iceland	GOD 7.4 km	NA	1	Magmatic	10	.2	0/1
**Holuhraun**	Iceland	VONK 50 km	NA	1	Magmatic	14	.5	0/1
**Ontake**	Japan	ONTA 2 km	NA	1	Phreatic	14	1.5	1/1
**Cordon Caulle**	Chile	PHU 10 km	TC	1		11	1	0/1
**Kawah Ijen**	Indonesia	POS 1 km	ID	1	Phreatic	13	.5	-
**Copahue**	Chile/ Argentina	COP 4.5 km		3	Phreatic	20 (3)	.8	3/3

Supplementary Figures

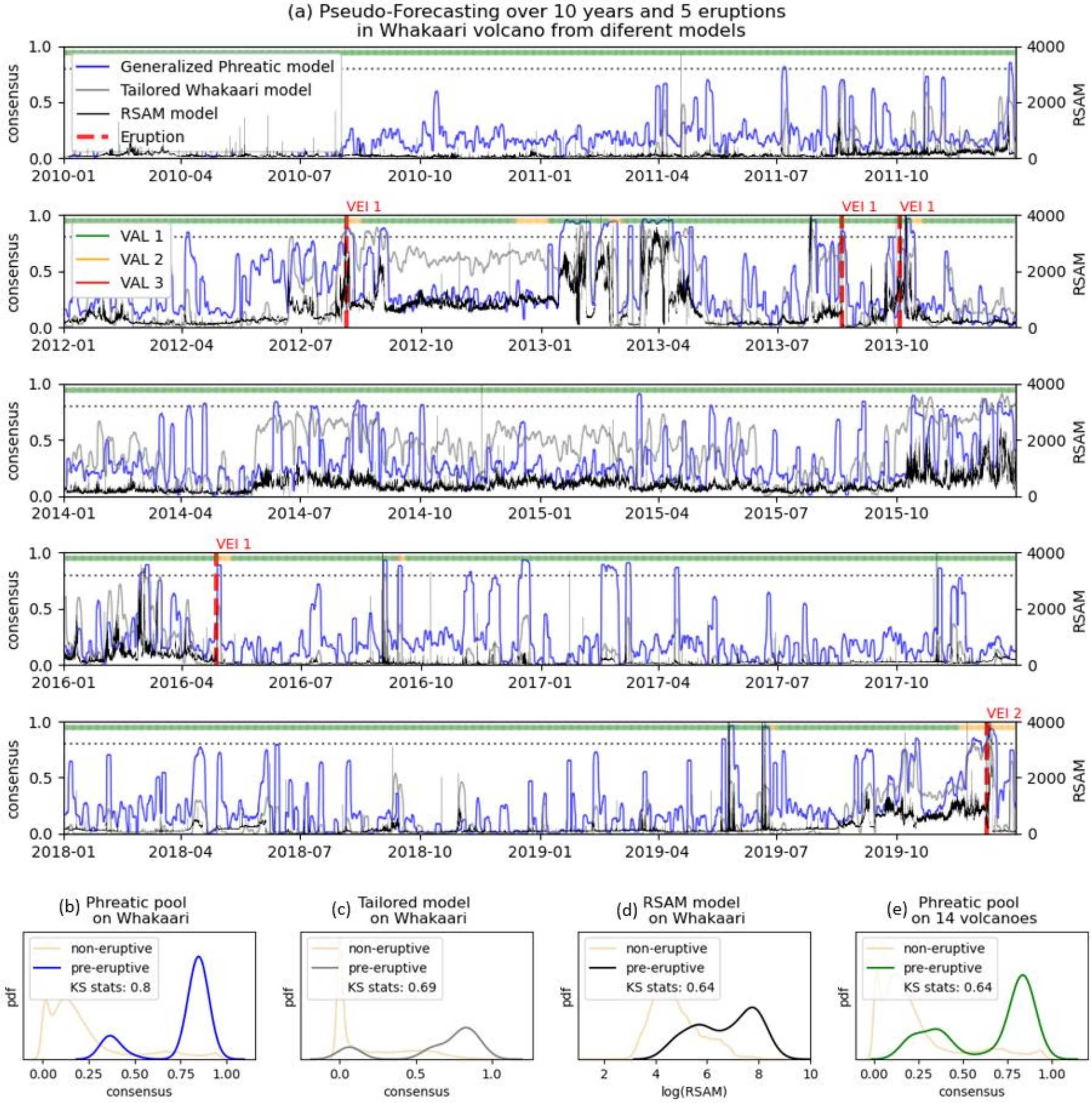

Figure S1. Forecast over the whole Whakaari record. Forecast models are indicated in the legend (eruption times by the red line). The forecasts correspond to the models tested on data that were not included in the training (out-of-sample). (b-e) Histograms display the consensus values of the two ML models, distinguishing between 2-days pre-eruptive values and non-eruptive values. Values are computed every 10-min. All values considered are out-of-sample. In (d), the same distributions are shown for RSAM values. Please note that pre-eruptive values encompass a period of 2 days before the reported eruption times, while non-eruptive values cover all days except those within 30 days around the reported eruption times.

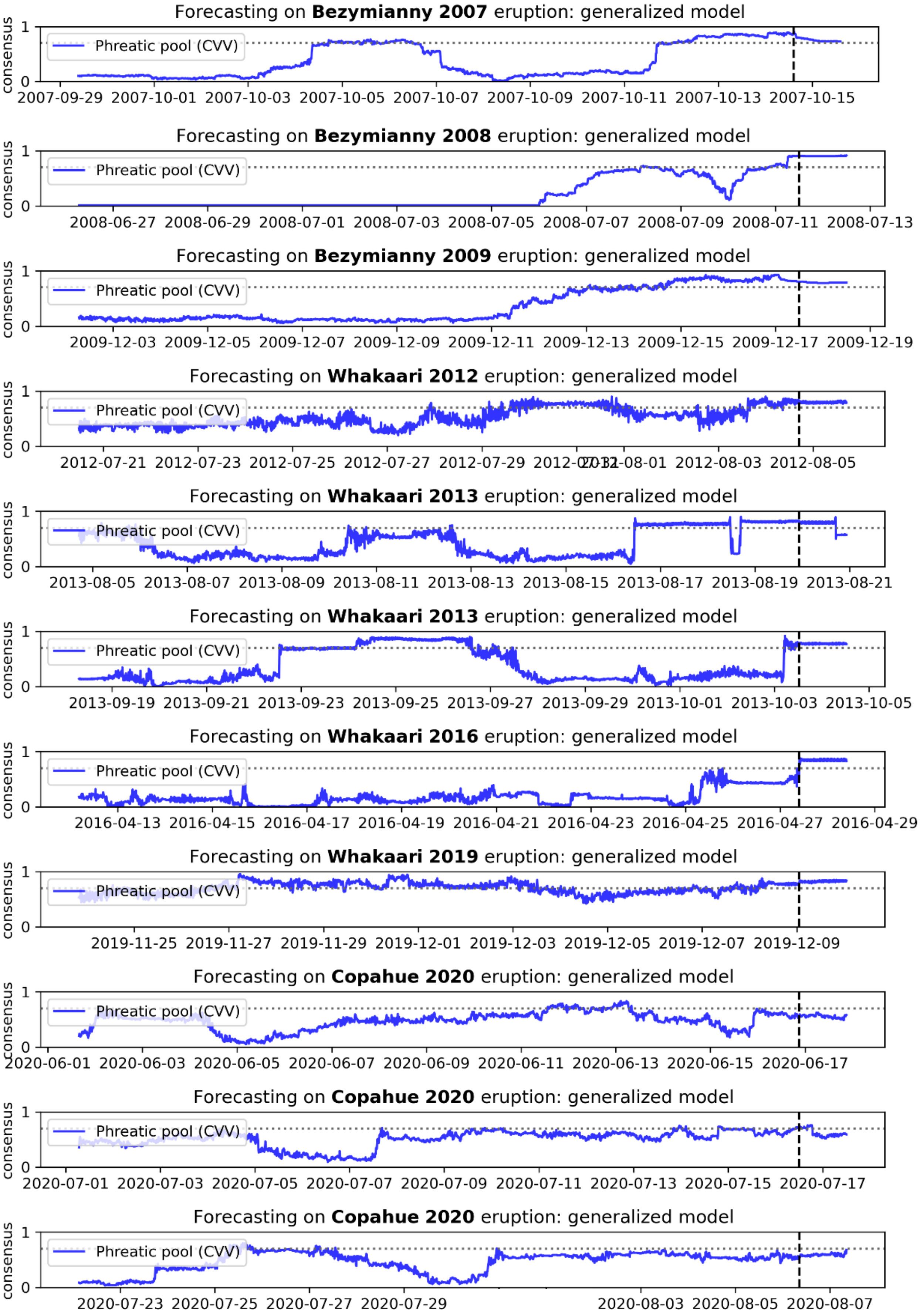

Figure S2. Forecast using phreatic generalized model trained on CVV in Whakaari and Bezymianny (pools are indicated in the legend, eruptions are indicated in the titles, and eruption times by the black dash line). The forecasts correspond to the models tested on eruptions that were not included in the training (out-of-sample). A reference threshold of .7 is indicated with the dash line. CVV and CVE that correspond to cross validation testing strategies are detailed in Figure S10.

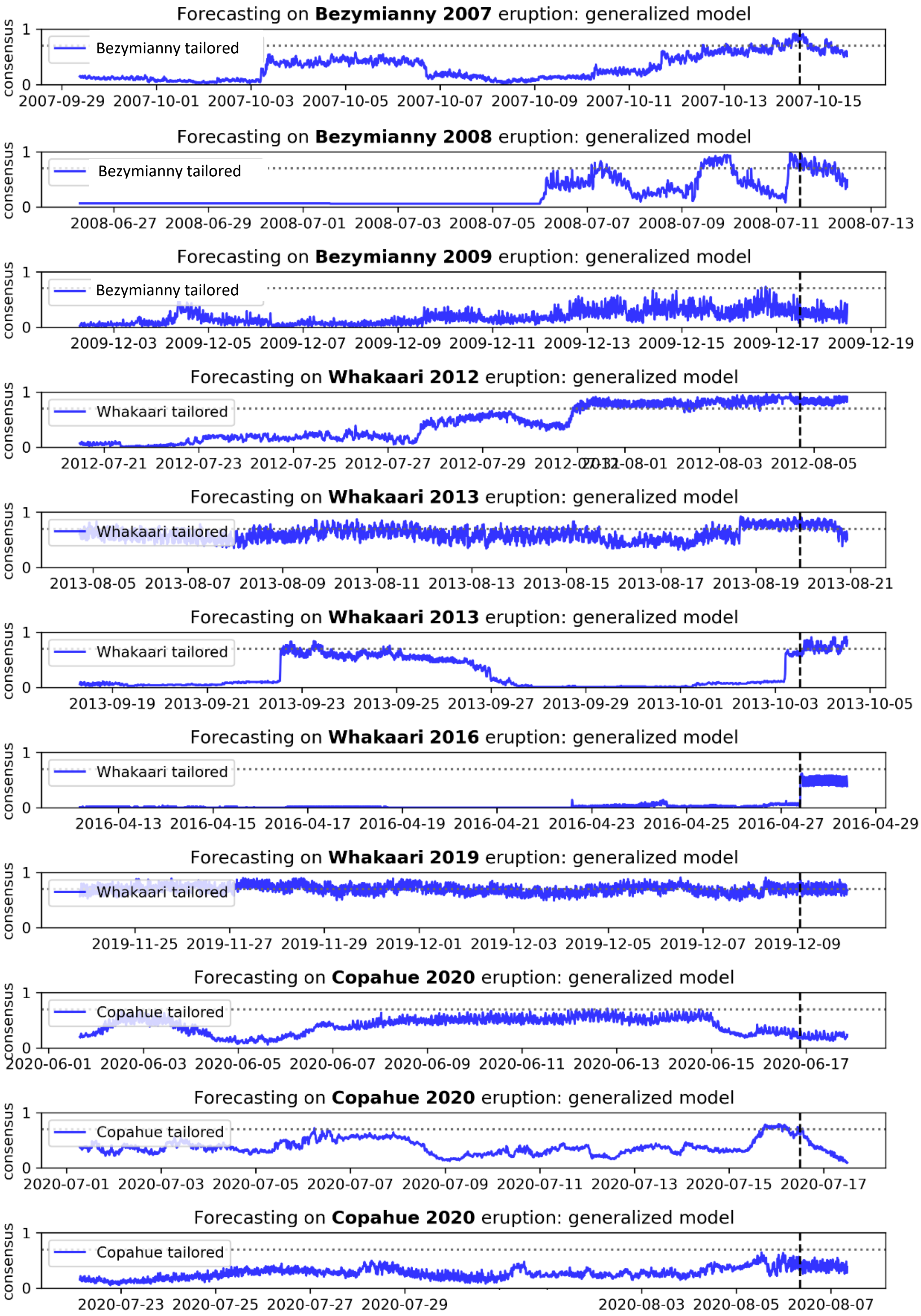

Figure S3. Forecast using tailored models prior to 13 eruptions in Whakaari, Bezymianny and Copahue (pools are indicated in the legend, eruptions are indicated in the titles, and eruption times by the black dash line). The forecasts correspond to the models tested on eruptions

that were not included in the training (out-of-sample). A reference threshold of .7 is indicated with the dash line. CVV and CVE that correspond to cross validation testing strategies are detailed in Figure S10.

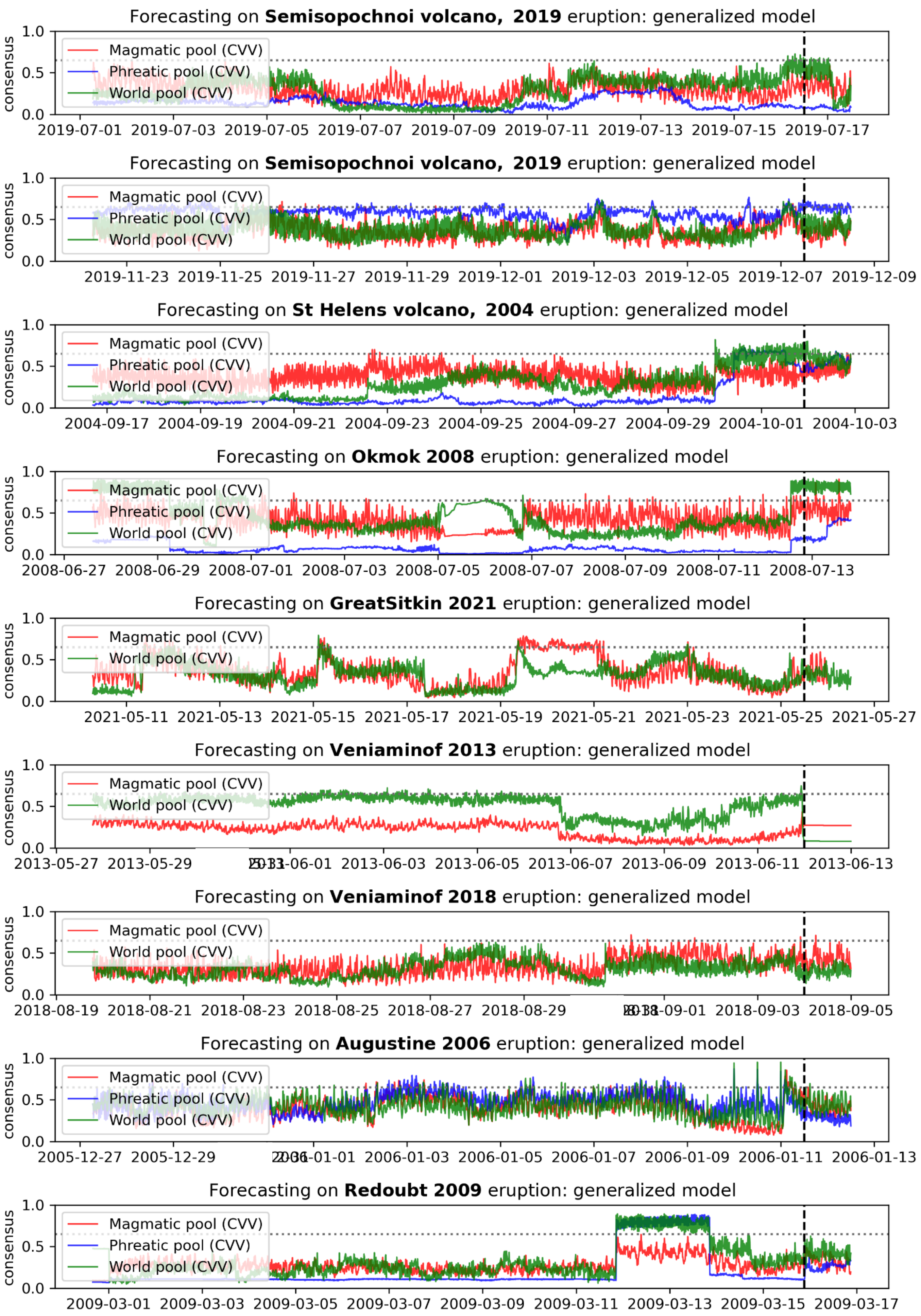
Forecasting on **Semisopochnoi volcano, 2019** eruption: generalized model
consensus
Magmatic pool (CVV)
Phreatic pool (CVV)
World pool (CVV)
2019-07-01 2019-07-03 2019-07-05 2019-07-07 2019-07-09 2019-07-11 2019-07-13 2019-07-15 2019-07-17
Forecasting on **Semisopochnoi volcano, 2019** eruption: generalized model
consensus
Magmatic pool (CVV)
Phreatic pool (CVV)
World pool (CVV)
2019-11-23 2019-11-25 2019-11-27 2019-11-29 2019-12-01 2019-12-03 2019-12-05 2019-12-07 2019-12-09
Forecasting on **St Helens volcano, 2004** eruption: generalized model
consensus
Magmatic pool (CVV)
Phreatic pool (CVV)
World pool (CVV)
2004-09-17 2004-09-19 2004-09-21 2004-09-23 2004-09-25 2004-09-27 2004-09-29 2004-10-01 2004-10-03
Forecasting on **Okmok 2008** eruption: generalized model
consensus
Magmatic pool (CVV)
Phreatic pool (CVV)
World pool (CVV)
2008-06-27 2008-06-29 2008-07-01 2008-07-03 2008-07-05 2008-07-07 2008-07-09 2008-07-11 2008-07-13
Forecasting on **GreatSitkin 2021** eruption: generalized model
consensus
Magmatic pool (CVV)
World pool (CVV)
2021-05-11 2021-05-13 2021-05-15 2021-05-17 2021-05-19 2021-05-21 2021-05-23 2021-05-25 2021-05-27
Forecasting on **Veniaminof 2013** eruption: generalized model
consensus
Magmatic pool (CVV)
World pool (CVV)
2013-05-27 2013-05-29 2013-06-03 2013-06-05 2013-06-07 2013-06-09 2013-06-11 2013-06-13
Forecasting on **Veniaminof 2018** eruption: generalized model
consensus
Magmatic pool (CVV)
World pool (CVV)
2018-08-19 2018-08-21 2018-08-23 2018-08-25 2018-08-27 2018-08-29 2018-09-03 2018-09-05
Forecasting on **Augustine 2006** eruption: generalized model
consensus
Magmatic pool (CVV)
Phreatic pool (CVV)
World pool (CVV)
2005-12-27 2005-12-29 2006-01-03 2006-01-05 2006-01-07 2006-01-09 2006-01-11 2006-01-13
Forecasting on **Redoubt 2009** eruption: generalized model
consensus
Magmatic pool (CVV)
Phreatic pool (CVV)
World pool (CVV)
2009-03-01 2009-03-03 2009-03-05 2009-03-07 2009-03-09 2009-03-11 2009-03-13 2009-03-15 2009-03-17

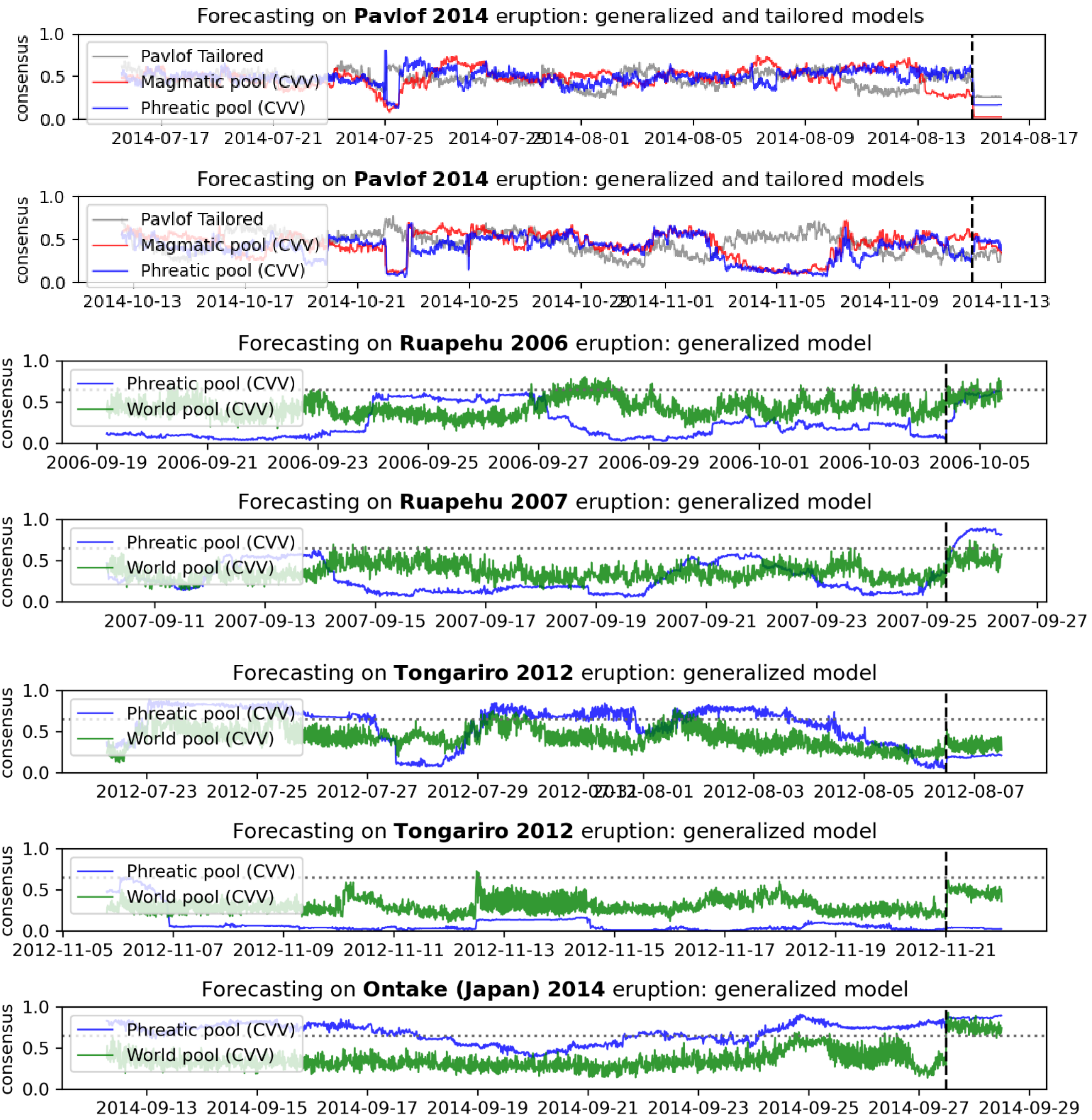

Figure S4. Forecast using generalized models trained on CVV prior to 14 eruptions (pools are indicated in the legend, eruptions are indicated in the titles, and eruption times by the black dash line). The forecasts correspond to the models tested on eruptions that were not included in the training (out-of-sample). CVV and CVE that correspond to cross validation testing strategies are detailed in Figure S10.

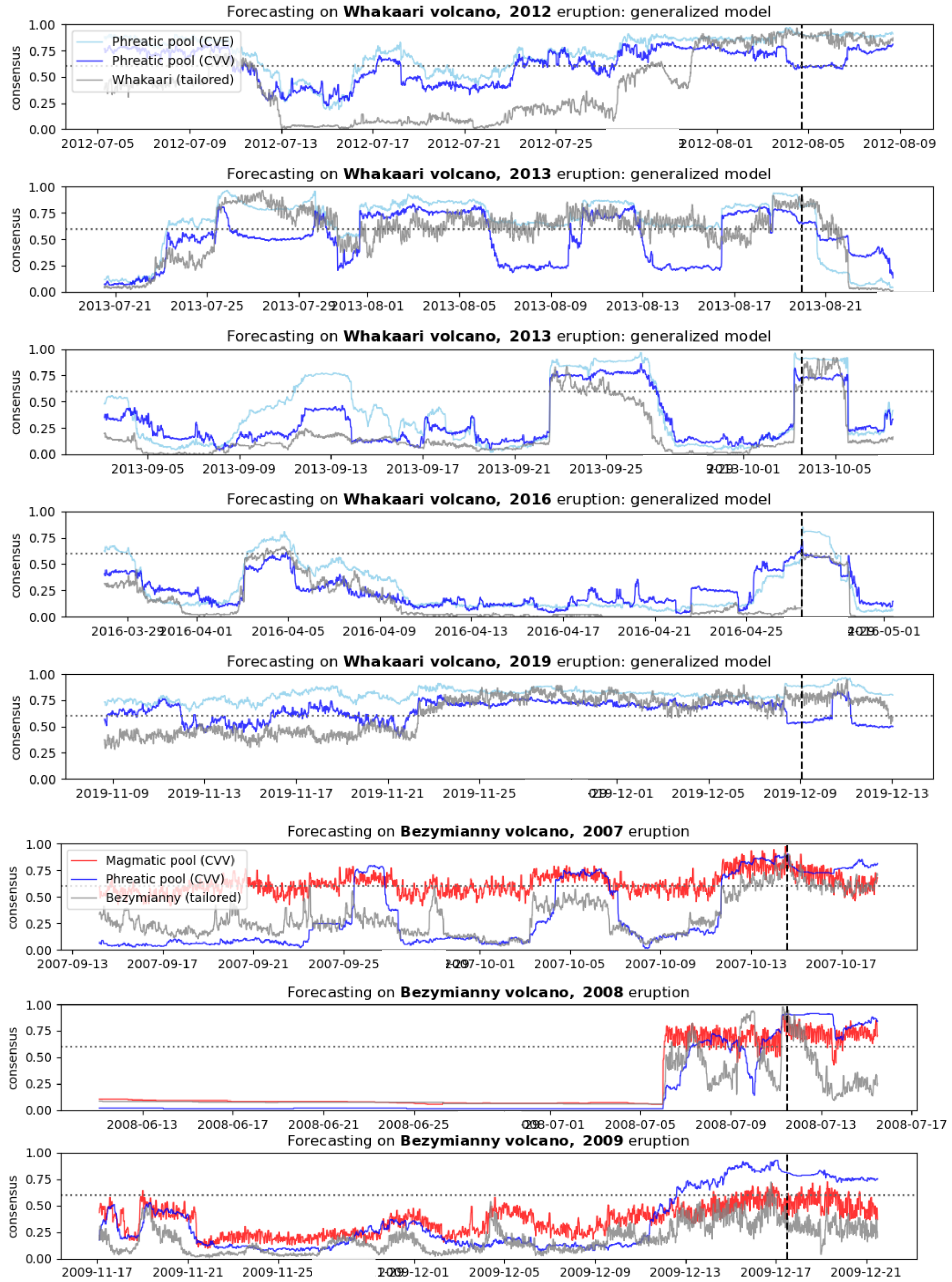

Figure S5. Forecast for different models prior to six eruptions considered in the Whakaari and Bezymianny eruptions (pools are indicated in the legend, eruptions are indicated in the titles, and eruption times by the black dash line). The forecasts correspond to the models tested on eruptions that were not included in the training (out-of-sample). This value is arbitrary, and the performance metrics of the models (described

in the next figures) are calculated for one hundred thresholds in the range [0, 1]. CVV and CVE that correspond to cross validation testing strategies are detailed in Figure S10.

Figure from: *Dempsey, D. E., Kempa-Liehr, A. W., Ardid, A, ... & Cronin, S. J. (2022). Evaluation of short-term probabilistic eruption forecasting at Whakaari, New Zealand. Bulletin of Volcanology, 84(10), 91.*

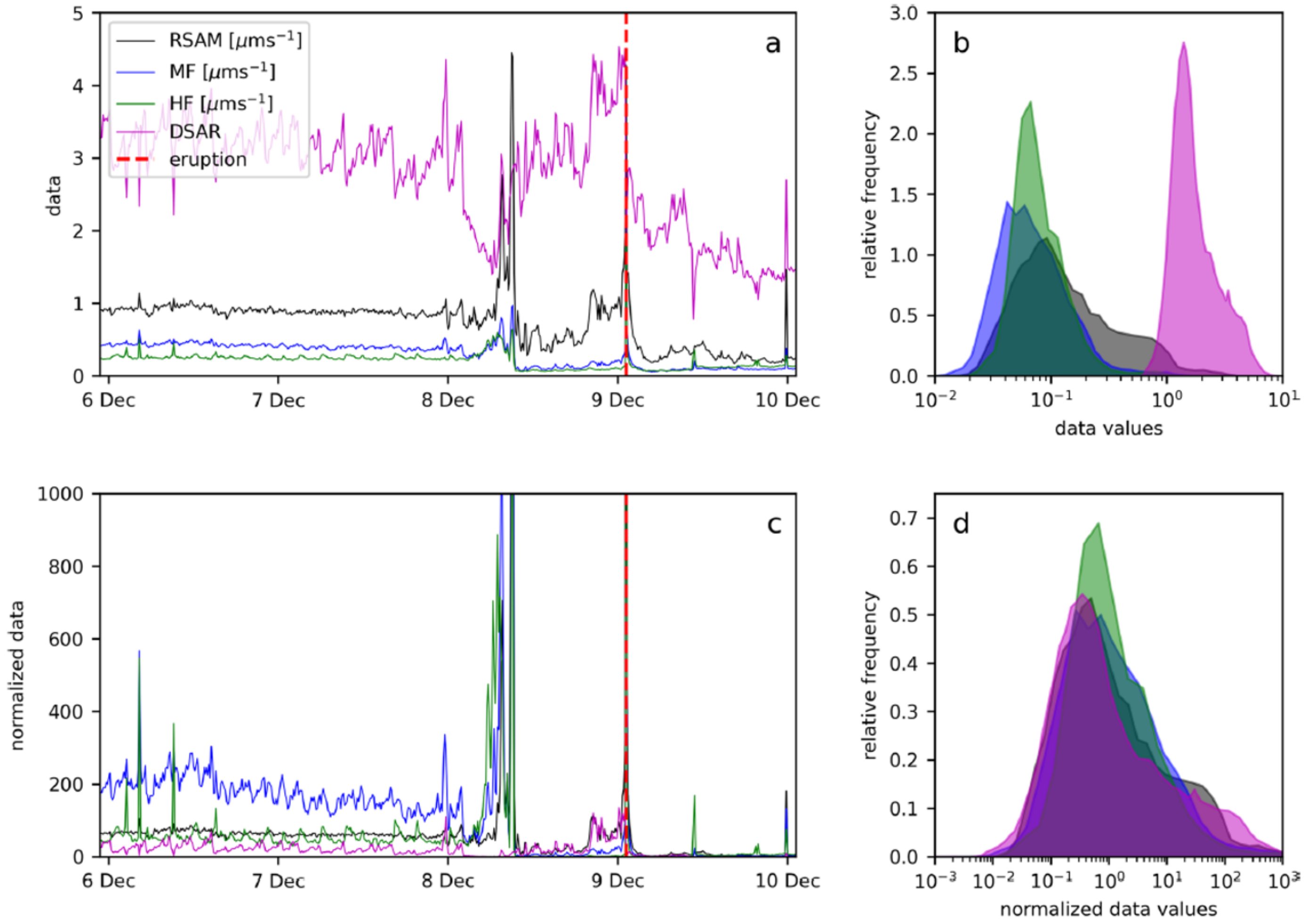

Figure S6. The figure is extracted from Dempsey et al., 2022, and it illustrates the effects of z-score normalization on the 10 years of Whakaari record across the four data streams RSAM, MF, HF, and DSAR. (a) Data normalization of input: Time series data before and after the 2019 eruption (indicated by the red dashed line): RSAM (black), MF (blue), HF (green), DSAR (magenta). (b) Distribution histograms displaying all data values throughout the 10.5-year study period. (c) Time series data normalized across the same time span as in (a). (d) Distribution histograms of data post z-score normalization.

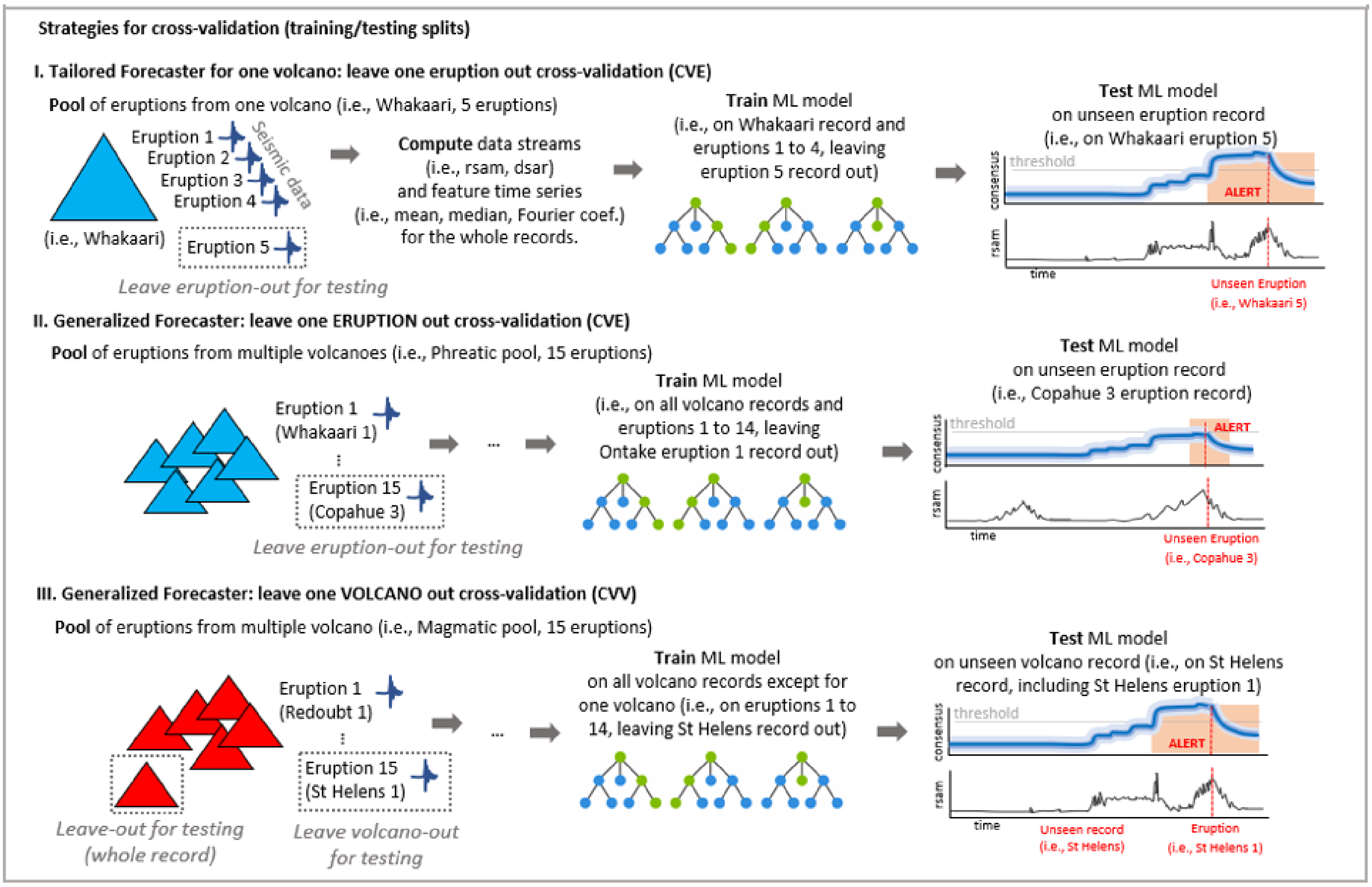

Figure S7. Cross-validation testing strategy explored in this study. (I) Cross-validation testing strategy for tailored forecasting models (single volcano with multiple eruptions), named here CVE (leave one eruption out). For the generalized model where, multiple volcanoes are used in the training, we explored two strategies: (II) CVE for generalized forecasters, where only data around eruptions are left out during training (but non-eruptive data from the target volcano is included); and (III) Cross-Validation leave on Volcano out (CVV) where the all the data from the target volcano is left out. This last strategy is the one presented in the main test, as it is more significant to the test for ergodicity, and it is the most generalized case.
