# Supplementary material for: Ergodic seismic precursors and transfer learning for short term eruption forecasting at data scarce volcanoes
Source: Nat Commun. 2025 Feb 25;16:1758. doi: 10.1038/s41467-025-56689-x (PMC11861682; doi:10.1038/s41467-025-56689-x)
Supplement: Supplementary file 2 — Description of Additional Supplementary Files [file 41467_2025_56689_MOESM2_ESM.pdf]

Description of Additional Supplementary Files

File name: Supplementary Data 1
Description: This dataset contains pre-processed seismic data used for developing and testing generalized eruption forecasting models. The data includes amplitude measurements sampled every 10 minutes for volcanoes in the study catalog. Raw waveform data for New Zealand volcanoes can be accessed from GEONET, and for Alaskan volcanoes, from IRIS. Both datasets are operable through the FDSN web service (https://www.fdsn.org/networks/). The provided dataset is intended for non-commercial research purposes. Users are encouraged to cite this article when utilizing this dataset.

File name: Supplementary Code 1
Description: This repository contains the codebase for the eruption forecasting study. It includes the main library for forecasting, named puia (meaning "volcanoes" in Māori), along with the following scripts:

A generalized machine learning forecaster that trains models using data from multiple volcanoes.
A test for a simple RSAM-based forecaster.
A script to compute Receiver Operating Characteristic (ROC) curves for model evaluation.
The provided example script demonstrates the implementation of a generalized forecasting model, using Whakaari for training and Bezymianny for testing. Key functionalities include:

Pre-processing seismic data streams (e.g., RSAM, MF, HF, DSAR).
Excluding eruption periods to avoid overfitting.
Training and evaluating models using a multi-volcano setup.
Generating high-resolution eruption forecasts with customizable parameters.
The code is provided under the Creative Commons Attribution-NonCommercial (CC BY-NC) License and is intended for non-commercial research purposes. Users are encouraged to report any bugs or issues via the GitHub repository. Detailed documentation and guidance on adapting the code to other volcanoes or use cases are included in the repository.
